# (*E*)-1-Phenyl­butan-2-one (2,4-dinitro­phen­yl)hydrazone

**DOI:** 10.1107/S1600536809041178

**Published:** 2009-10-17

**Authors:** Carlos F. R. A. C. Lima, Ligia R. Gomes, Luís M. N. B. F. Santos, José E. Rodriguez-Borges, John Nicolson Low

**Affiliations:** a Centro de Investigação em Química, Departamento de Química, Faculdade de Ciências, Universidade do Porto, Rua do Campo Alegre, 687, P-4169_007 Porto, Portugal; b REQUIMTE, Departamento de Química, Faculdade de Ciências, Universidade do Porto, Rua do Campo Alegre, 687, P-4169_007 Porto, Portugal; c Department of Chemistry, University of Aberdeen, Meston Walk, Old Aberdeen AB24 3UE, Scotland.

## Abstract

In the title compound, C_16_H_16_N_4_O_4_, the dihedral angle between the aromatic rings is 79.04 (8)° and an intra­molecular N—H⋯O hydrogen bond occurs. In the crystal, weak C—H.·O and C—H..π inter­actions link the mol­ecules, forming sheets.

## Related literature

For the structure of the related 2,4-dinitro­phenyl hydrazine, see: Wardell *et al.* (2006 ▶). For graph-set notation, see: Bernstein *et al.* (1995 ▶).
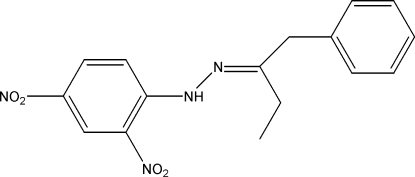


## Experimental

### 

#### Crystal data

C_16_H_16_N_4_O_4_
*M* *_r_* = 328.33
Monoclinic, 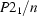

*a* = 15.8919 (13) Å
*b* = 4.9446 (3) Å
*c* = 20.7397 (17) Å
β = 105.267 (5)°
*V* = 1572.2 (2) Å^3^
*Z* = 4
Mo *K*α radiation
μ = 0.10 mm^−1^
*T* = 120 K
0.30 × 0.05 × 0.02 mm

#### Data collection

Bruker SMART APEXII diffractometer
Absorption correction: multi-scan (*SADABS*; Bruker, 2004 ▶) *T*_min_ = 0.970, *T*_max_ = 0.998
14305 measured reflections
3531 independent reflections
2418 reflections with *I* > 2σ(*I*)
*R*_int_ = 0.048

#### Refinement

*R*[*F* ^2^ > 2σ(*F* ^2^)] = 0.041
*wR*(*F* ^2^) = 0.101
*S* = 1.01
3531 reflections
218 parameters
1 restraint
H-atom parameters constrained
Δρ_max_ = 0.25 e Å^−3^
Δρ_min_ = −0.19 e Å^−3^

### 

Data collection: *APEX2* (Bruker, 2004 ▶); cell refinement: *APEX2* and *SAINT* (Bruker, 2004 ▶); data reduction: *SAINT*; program(s) used to solve structure: *SHELXS97* (Sheldrick, 2008 ▶); program(s) used to refine structure: *SHELXL97* (Sheldrick, 2008 ▶); molecular graphics: *ORTEPII* (Johnson, 1976 ▶) and *PLATON* (Spek, 2009 ▶); software used to prepare material for publication: *SHELXL97*.

## Supplementary Material

Crystal structure: contains datablocks global, I. DOI: 10.1107/S1600536809041178/hb5130sup1.cif

Structure factors: contains datablocks I. DOI: 10.1107/S1600536809041178/hb5130Isup2.hkl

Additional supplementary materials:  crystallographic information; 3D view; checkCIF report

## Figures and Tables

**Table 1:** Hydrogen-bond geometry (Å, °)

*D*—H⋯*A*	*D*—H	H⋯*A*	*D*⋯*A*	*D*—H⋯*A*
N1—H1⋯O122	0.85	1.95	2.5966 (17)	132
C3—H3*A*⋯O142^i^	0.99	2.50	3.432 (2)	158
C32—H32⋯O142^i^	0.95	2.52	3.349 (2)	146
C3—H3*B*⋯*Cg*2^ii^	0.99	2.75	3.534 (2)	136

Symmetry codes: (i) 
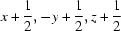
; (ii) 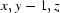. *Cg*2 is the centroid of C31–C36 ring.

**Fig. 1.**
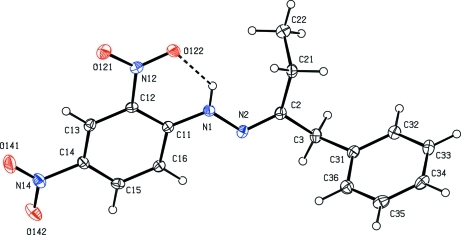
A view of (I) with displacement ellipsoids drawn at the 30% probability level.

**Fig. 2.**
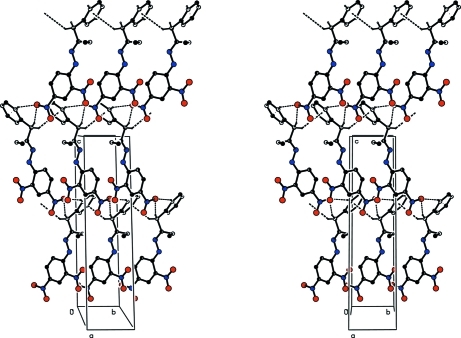
A stereoview of part of the crystal structure of (I), showing part of the sheet formed by the C—H..O and C—H···π interactions. Hydrogen atoms not involved in the motifs are not included.

## supplementary crystallographic information

### Comment

The molecular geometry and conformation is as expected taking acount of electronic repulsions and steric effects. The orange colour is caused by the conjugation of the nitrophenyl with the –N—N= group. The backbone of the molecule is essentially planar with only the methyl group, C21, and the phenyl group attached to C3 lying out of the plane of the molecule, Fig. 1.

Atom N1 forms an intramolecular hydrogen bond *via* H1 with atom O122 so forming an *R*(6) ring, (Bernstein *et al.*, 1995). Atom O142 acts as a hydrogen bond acceptor from donor atoms C3, *via* H3B, and C32, *via* H32, thus forming an *R*^2^_1_(5) ring which links screw-related molecules into chains running parallel to (101). These chains are linked to form sheets running parallel to the *b* axis by the C—H···π contact in which C3 acts as a donor atom *via* H3B to the phenyl ring containing C31, Fig.2.

There is a short intermolecular nitro to nitro group contact between O122···N12(-*x* + 1.5, *y* + 1/2, -*z* + 1/2) of 2.76 Å.

The relevant bonds and angles compare well with those in 2,4-dinitrophenylhydrazine: Wardell *et al.* 2006.

### Experimental

The title compound was obtained from the condensation reaction of 1-phenyl-2-butanone with dinitrophenylhydrazine. The liquid 1-phenyl-2-butanone (2.0 mmol) was added to a warm solution (323 K) of 2,4-dinitrophenylhydrazine (2.5 mmol) in a mixture of 10 ml of ethanol/ 1 ml of HCl (37%) and allowed to react for 30 minutes. The resulting mixture was extracted with ethylacetate. A solid product was obtained after evaporation of the solvent this was first re-crystallized with ethanol and then with ethylacetate. Slow evaporation of a dichloromethane solution gave orange needles of (I).

### Refinement

H atoms were treated as riding atoms with C—H(aromatic), 0.95 Å, C—H2(aliphatic),0.99 Å, *C*—*H*(methyl), 0.98 Å. The H atom attached to N1 was located on a difference map, fixed to 0.85 Å, and then refined as a riding atom. The reflections 101 and 101 were omitted from the refinement as they were obscured by the beamstop.

### Crystal data

**Table:**

C_16_H_16_N_4_O_4_	*F*(000) = 688
*M**_r_* = 328.33	*D*_x_ = 1.387 Mg m^−^^3^
Monoclinic, *P*2_1_/*n*	Melting point: not measured K
Hall symbol: -P 2yn	Mo *K*α radiation, λ = 0.71073 Å
*a* = 15.8919 (13) Å	Cell parameters from 2030 reflections
*b* = 4.9446 (3) Å	θ = 3.1–26.5°
*c* = 20.7397 (17) Å	µ = 0.10 mm^−^^1^
β = 105.267 (5)°	*T* = 120 K
*V* = 1572.2 (2) Å^3^	Needle, orange
*Z* = 4	0.30 × 0.05 × 0.02 mm

### Data collection

**Table:**

Bruker SMART APEX diffractometer	3531 independent reflections
Radiation source: fine-focus sealed tube	2418 reflections with *I* > 2σ(*I*)
graphite	*R*_int_ = 0.048
Detector resolution: 8.333 pixels mm^-1^	θ_max_ = 27.3°, θ_min_ = 1.5°
ω scans	*h* = −20→20
Absorption correction: multi-scan (*SADABS*; Bruker, 2004)	*k* = −5→6
*T*_min_ = 0.970, *T*_max_ = 0.998	*l* = −26→26
14305 measured reflections	

### Refinement

**Table:**

Refinement on *F*^2^	Secondary atom site location: difference Fourier map
Least-squares matrix: full	Hydrogen site location: inferred from neighbouring sites
*R*[*F*^2^ > 2σ(*F*^2^)] = 0.041	H-atom parameters constrained
*wR*(*F*^2^) = 0.101	*w* = 1/[σ^2^(*F*_o_^2^) + (0.037*P*)^2^ + 0.4505*P*] where *P* = (*F*_o_^2^ + 2*F*_c_^2^)/3
*S* = 1.01	(Δ/σ)_max_ < 0.001
3531 reflections	Δρ_max_ = 0.25 e Å^−^^3^
218 parameters	Δρ_min_ = −0.19 e Å^−^^3^
1 restraint	Extinction correction: *SHELXL97* (Sheldrick, 2008), Fc^*^=kFc[1+0.001xFc^2^λ^3^/sin(2θ)]^-1/4^
Primary atom site location: structure-invariant direct methods	Extinction coefficient: 0.0044 (11)

### Special details

**Table:**

Geometry. All e.s.d.'s (except the e.s.d. in the dihedral angle between two l.s. planes) are estimated using the full covariance matrix. The cell e.s.d.'s are taken into account individually in the estimation of e.s.d.'s in distances, angles and torsion angles; correlations between e.s.d.'s in cell parameters are only used when they are defined by crystal symmetry. An approximate (isotropic) treatment of cell e.s.d.'s is used for estimating e.s.d.'s involving l.s. planes.
Refinement. Refinement of *F*^2^ against ALL reflections. The weighted *R*-factor *wR* and goodness of fit *S* are based on *F*^2^, conventional *R*-factors *R* are based on *F*, with *F* set to zero for negative *F*^2^. The threshold expression of *F*^2^ > σ(*F*^2^) is used only for calculating *R*-factors(gt) *etc*. and is not relevant to the choice of reflections for refinement. *R*-factors based on *F*^2^ are statistically about twice as large as those based on *F*, and *R*- factors based on ALL data will be even larger.

### Fractional atomic coordinates and isotropic or equivalent isotropic displacement parameters (Å^2^)

**Table:**

	*x*	*y*	*z*	*U*_iso_*/*U*_eq_	
O121	0.65610 (8)	0.8996 (2)	0.17034 (6)	0.0325 (3)	
O122	0.70928 (7)	0.9899 (2)	0.27500 (6)	0.0276 (3)	
O141	0.44704 (9)	0.2157 (3)	0.09414 (6)	0.0454 (4)	
O142	0.41347 (8)	−0.0583 (3)	0.16477 (7)	0.0427 (4)	
N1	0.66460 (8)	0.7090 (3)	0.36646 (6)	0.0225 (3)	
H1	0.6995	0.8261	0.3582	0.027*	
N2	0.66485 (9)	0.6313 (3)	0.43054 (6)	0.0225 (3)	
N12	0.66156 (9)	0.8554 (3)	0.22955 (7)	0.0225 (3)	
N14	0.45198 (9)	0.1408 (3)	0.15136 (7)	0.0301 (3)	
C2	0.71757 (10)	0.7597 (3)	0.47833 (8)	0.0220 (3)	
C3	0.71901 (11)	0.6653 (3)	0.54758 (8)	0.0250 (4)	
H3A	0.7789	0.6066	0.5708	0.030*	
H3B	0.6800	0.5068	0.5442	0.030*	
C11	0.61365 (10)	0.5756 (3)	0.31389 (7)	0.0195 (3)	
C12	0.61004 (10)	0.6396 (3)	0.24669 (8)	0.0194 (3)	
C13	0.55682 (10)	0.4984 (3)	0.19381 (8)	0.0228 (4)	
H13	0.5557	0.5436	0.1491	0.027*	
C14	0.50597 (10)	0.2934 (3)	0.20667 (8)	0.0227 (4)	
C15	0.50676 (10)	0.2231 (3)	0.27206 (8)	0.0235 (4)	
H15	0.4710	0.0798	0.2801	0.028*	
C16	0.55915 (10)	0.3614 (3)	0.32419 (8)	0.0223 (4)	
H16	0.5592	0.3133	0.3686	0.027*	
C21	0.77979 (11)	0.9796 (3)	0.47154 (8)	0.0269 (4)	
H21A	0.7905	1.0997	0.5111	0.032*	
H21B	0.7532	1.0896	0.4315	0.032*	
C22	0.86642 (12)	0.8638 (4)	0.46542 (10)	0.0410 (5)	
H22A	0.9053	1.0120	0.4607	0.061*	
H22B	0.8560	0.7461	0.4261	0.061*	
H22C	0.8936	0.7587	0.5056	0.061*	
C31	0.69053 (11)	0.8820 (3)	0.58860 (8)	0.0228 (4)	
C32	0.74933 (11)	1.0031 (3)	0.64209 (8)	0.0257 (4)	
H32	0.8088	0.9494	0.6534	0.031*	
C33	0.72226 (12)	1.2021 (3)	0.67932 (8)	0.0300 (4)	
H33	0.7633	1.2831	0.7160	0.036*	
C34	0.63628 (12)	1.2830 (4)	0.66341 (9)	0.0317 (4)	
H34	0.6179	1.4195	0.6890	0.038*	
C35	0.57701 (12)	1.1645 (4)	0.61015 (9)	0.0332 (4)	
H35	0.5177	1.2198	0.5989	0.040*	
C36	0.60383 (11)	0.9647 (4)	0.57296 (9)	0.0300 (4)	
H36	0.5626	0.8836	0.5365	0.036*	

### Atomic displacement parameters (Å^2^)

**Table:**

	*U*^11^	*U*^22^	*U*^33^	*U*^12^	*U*^13^	*U*^23^
O121	0.0417 (8)	0.0314 (7)	0.0260 (6)	−0.0003 (6)	0.0118 (6)	0.0090 (5)
O122	0.0283 (7)	0.0230 (6)	0.0326 (7)	−0.0063 (5)	0.0100 (5)	−0.0043 (5)
O141	0.0493 (9)	0.0554 (9)	0.0250 (7)	−0.0098 (7)	−0.0019 (6)	−0.0049 (6)
O142	0.0380 (8)	0.0326 (7)	0.0493 (8)	−0.0125 (6)	−0.0030 (6)	−0.0037 (6)
N1	0.0242 (7)	0.0225 (7)	0.0210 (7)	−0.0041 (6)	0.0067 (6)	0.0003 (6)
N2	0.0256 (7)	0.0230 (7)	0.0191 (7)	0.0027 (6)	0.0063 (6)	0.0008 (6)
N12	0.0228 (7)	0.0203 (7)	0.0256 (7)	0.0040 (6)	0.0086 (6)	0.0033 (6)
N14	0.0232 (8)	0.0291 (8)	0.0331 (8)	0.0018 (6)	−0.0011 (6)	−0.0049 (7)
C2	0.0230 (8)	0.0197 (8)	0.0231 (8)	0.0056 (7)	0.0058 (7)	−0.0023 (7)
C3	0.0298 (9)	0.0224 (8)	0.0212 (8)	0.0029 (7)	0.0040 (7)	0.0004 (7)
C11	0.0188 (8)	0.0171 (8)	0.0226 (8)	0.0040 (6)	0.0056 (6)	−0.0013 (6)
C12	0.0184 (8)	0.0167 (8)	0.0246 (8)	0.0021 (6)	0.0082 (6)	0.0017 (7)
C13	0.0227 (9)	0.0248 (9)	0.0213 (8)	0.0060 (7)	0.0066 (7)	0.0008 (7)
C14	0.0187 (8)	0.0210 (8)	0.0267 (8)	0.0020 (7)	0.0028 (7)	−0.0057 (7)
C15	0.0205 (8)	0.0188 (8)	0.0327 (9)	−0.0007 (7)	0.0097 (7)	−0.0003 (7)
C16	0.0237 (9)	0.0215 (8)	0.0230 (8)	0.0015 (7)	0.0085 (7)	0.0006 (7)
C21	0.0311 (10)	0.0275 (9)	0.0217 (8)	−0.0036 (7)	0.0063 (7)	−0.0053 (7)
C22	0.0344 (11)	0.0536 (13)	0.0383 (10)	−0.0071 (9)	0.0157 (9)	−0.0107 (10)
C31	0.0286 (9)	0.0205 (8)	0.0204 (8)	0.0025 (7)	0.0084 (7)	0.0044 (7)
C32	0.0272 (9)	0.0245 (9)	0.0246 (8)	0.0035 (7)	0.0056 (7)	0.0028 (7)
C33	0.0404 (11)	0.0275 (9)	0.0219 (8)	−0.0012 (8)	0.0081 (8)	−0.0024 (7)
C34	0.0413 (11)	0.0275 (10)	0.0323 (9)	0.0049 (8)	0.0206 (8)	−0.0001 (8)
C35	0.0288 (10)	0.0361 (10)	0.0382 (10)	0.0070 (8)	0.0149 (8)	0.0046 (9)
C36	0.0282 (10)	0.0329 (10)	0.0278 (9)	0.0005 (8)	0.0053 (7)	−0.0002 (8)

### Geometric parameters (Å, °)

**Table:**

O121—N12	1.2276 (16)	C15—C16	1.362 (2)
O122—N12	1.2370 (17)	C15—H15	0.9500
O141—N14	1.2258 (18)	C16—H16	0.9500
O142—N14	1.2292 (19)	C21—C22	1.527 (2)
N1—C11	1.3469 (19)	C21—H21A	0.9900
N1—N2	1.3823 (17)	C21—H21B	0.9900
N1—H1	0.85	C22—H22A	0.9800
N2—C2	1.284 (2)	C22—H22B	0.9800
N12—C12	1.445 (2)	C22—H22C	0.9800
N14—C14	1.450 (2)	C31—C32	1.385 (2)
C2—C21	1.501 (2)	C31—C36	1.391 (2)
C2—C3	1.505 (2)	C32—C33	1.387 (2)
C3—C31	1.510 (2)	C32—H32	0.9500
C3—H3A	0.9900	C33—C34	1.378 (2)
C3—H3B	0.9900	C33—H33	0.9500
C11—C12	1.416 (2)	C34—C35	1.379 (3)
C11—C16	1.420 (2)	C34—H34	0.9500
C12—C13	1.385 (2)	C35—C36	1.388 (2)
C13—C14	1.366 (2)	C35—H35	0.9500
C13—H13	0.9500	C36—H36	0.9500
C14—C15	1.397 (2)		
			
C11—N1—N2	119.42 (13)	C15—C16—C11	121.65 (15)
C11—N1—H1	117.1	C15—C16—H16	119.2
N2—N1—H1	123.1	C11—C16—H16	119.2
C2—N2—N1	116.15 (13)	C2—C21—C22	111.49 (14)
O121—N12—O122	122.28 (13)	C2—C21—H21A	109.3
O121—N12—C12	118.84 (13)	C22—C21—H21A	109.3
O122—N12—C12	118.88 (13)	C2—C21—H21B	109.3
O141—N14—O142	123.53 (15)	C22—C21—H21B	109.3
O141—N14—C14	118.82 (15)	H21A—C21—H21B	108.0
O142—N14—C14	117.64 (15)	C21—C22—H22A	109.5
N2—C2—C21	126.70 (14)	C21—C22—H22B	109.5
N2—C2—C3	115.22 (14)	H22A—C22—H22B	109.5
C21—C2—C3	117.99 (14)	C21—C22—H22C	109.5
C2—C3—C31	112.72 (13)	H22A—C22—H22C	109.5
C2—C3—H3A	109.0	H22B—C22—H22C	109.5
C31—C3—H3A	109.0	C32—C31—C36	118.53 (15)
C2—C3—H3B	109.0	C32—C31—C3	121.28 (15)
C31—C3—H3B	109.0	C36—C31—C3	120.19 (15)
H3A—C3—H3B	107.8	C31—C32—C33	120.67 (16)
N1—C11—C12	123.19 (14)	C31—C32—H32	119.7
N1—C11—C16	120.26 (14)	C33—C32—H32	119.7
C12—C11—C16	116.54 (14)	C34—C33—C32	120.39 (17)
C13—C12—C11	121.67 (14)	C34—C33—H33	119.8
C13—C12—N12	116.42 (14)	C32—C33—H33	119.8
C11—C12—N12	121.91 (14)	C33—C34—C35	119.58 (16)
C14—C13—C12	119.28 (15)	C33—C34—H34	120.2
C14—C13—H13	120.4	C35—C34—H34	120.2
C12—C13—H13	120.4	C34—C35—C36	120.17 (17)
C13—C14—C15	121.32 (15)	C34—C35—H35	119.9
C13—C14—N14	119.28 (15)	C36—C35—H35	119.9
C15—C14—N14	119.38 (15)	C35—C36—C31	120.67 (17)
C16—C15—C14	119.54 (15)	C35—C36—H36	119.7
C16—C15—H15	120.2	C31—C36—H36	119.7
C14—C15—H15	120.2		
			
C11—N1—N2—C2	177.22 (14)	O142—N14—C14—C13	173.19 (15)
N1—N2—C2—C21	−1.4 (2)	O141—N14—C14—C15	174.98 (15)
N1—N2—C2—C3	−177.96 (12)	O142—N14—C14—C15	−5.0 (2)
N2—C2—C3—C31	−117.81 (16)	C13—C14—C15—C16	0.1 (2)
C21—C2—C3—C31	65.32 (19)	N14—C14—C15—C16	178.26 (14)
N2—N1—C11—C12	−179.33 (13)	C14—C15—C16—C11	−0.3 (2)
N2—N1—C11—C16	1.5 (2)	N1—C11—C16—C15	179.86 (14)
N1—C11—C12—C13	−179.94 (14)	C12—C11—C16—C15	0.6 (2)
C16—C11—C12—C13	−0.7 (2)	N2—C2—C21—C22	−86.2 (2)
N1—C11—C12—N12	0.1 (2)	C3—C2—C21—C22	90.25 (17)
C16—C11—C12—N12	179.29 (13)	C2—C3—C31—C32	−109.84 (17)
O121—N12—C12—C13	−0.6 (2)	C2—C3—C31—C36	70.0 (2)
O122—N12—C12—C13	179.04 (13)	C36—C31—C32—C33	0.1 (2)
O121—N12—C12—C11	179.42 (14)	C3—C31—C32—C33	179.95 (15)
O122—N12—C12—C11	−1.0 (2)	C31—C32—C33—C34	−0.2 (3)
C11—C12—C13—C14	0.5 (2)	C32—C33—C34—C35	0.1 (3)
N12—C12—C13—C14	−179.50 (13)	C33—C34—C35—C36	0.2 (3)
C12—C13—C14—C15	−0.2 (2)	C34—C35—C36—C31	−0.2 (3)
C12—C13—C14—N14	−178.37 (14)	C32—C31—C36—C35	0.1 (2)
O141—N14—C14—C13	−6.8 (2)	C3—C31—C36—C35	−179.73 (15)

### Hydrogen-bond geometry (Å, °)

**Table:**

*D*—H···*A*	*D*—H	H···*A*	*D*···*A*	*D*—H···*A*
N1—H1···O122	0.85	1.95	2.5966 (17)	132
C3—H3A···O142^i^	0.99	2.50	3.432 (2)	158
C32—H32···O142^i^	0.95	2.52	3.349 (2)	146
C3—H3B···Cg2^ii^	0.99	2.75	3.534 (2)	136

Symmetry codes: (i) *x*+1/2, −*y*+1/2, *z*+1/2; (ii) *x*, *y*−1, *z*.
